# The Take Control Course: Conceptual Rationale for the Development of a Transdiagnostic Group for Common Mental Health Problems

**DOI:** 10.3389/fpsyg.2016.00099

**Published:** 2016-02-10

**Authors:** Lydia Morris, Warren Mansell, Phil McEvoy

**Affiliations:** ^1^School of Psychological Sciences, University of ManchesterManchester, UK; ^2^Six Degrees Social Enterprise, CIC, The Angel CentreSalford, UK

**Keywords:** transdiagnostic, group intervention, anxiety, depression, primary care, control theory

## Abstract

**Background:** Increasingly, research supports the utility of a transdiagnostic understanding of psychopathology. However, there is no consensus regarding the theoretical approach that best explains this. Transdiagnostic interventions can offer service delivery advantages; this is explored in the current review, focusing on group modalities and primary care settings.

**Objective:** This review seeks to explore whether a Perceptual Control Theory (PCT) explanation of psychopathology across disorders is a valid one. Further, this review illustrates the process of developing a novel transdiagnostic intervention (Take Control Course; TCC) from a PCT theory of functioning.

**Method:** Narrative review.

**Results and Conclusions:** Considerable evidence supports key tenets of PCT. Further, PCT offers a novel perspective regarding the mechanisms by which a number of familiar techniques, such as exposure and awareness, are effective. However, additional research is required to directly test the relative contribution of some PCT mechanisms predicted to underlie psychopathology. Directions for future research are considered.

Cognitive Behavioral Therapy (CBT) offers a range of highly efficacious treatment protocols targeting specific disorders, as evidenced by a substantial number of RCTs (Butler et al., 2006; Hofmann et al., 2012). However, significant numbers of people do not receive evidence-based psychological interventions for psychological problems (Shafran et al., 2009; Castelnuovo, 2010; Hofmann, 2013); a “gap” between research findings and clinical practice (Shafran et al., 2009). Various explanations for this gap have been considered including: clinician beliefs that manualized treatments are not sufficiently flexible for routine practice; insufficient funding for training and interventions; and lack of knowledge of how to best convey and assess CBT skills (Shafran et al., 2009; Dobson and Beshai, 2013).

The plurality of disorder-specific protocols within the “CBT family” can provide challenges in disseminating CBT and could contribute to the gap between research findings and practice (Mansell, 2008; Dobson and Beshai, 2013; Hofmann, 2013). This provides an ongoing imperative for the development of interventions that develop generalizable clinician skills and are effective for clients with comorbid problems (Shafran et al., 2009; McHugh and Barlow, 2010). Transdiagnostic interventions may facilitate this process by providing interventions that are empirically grounded, but flexible to deliver and disseminate (McHugh et al., 2009; Wilamowska et al., 2010).

Firstly, this paper briefly reviews the rationale and empirical basis for a transdiagnostic approach. Secondly, Perceptual Control Theory (PCT; Powers, 1973) is detailed. PCT offers a conceptual underpinning for transdiagnostic CBT interventions (Mansell et al., 2012). Thirdly, the PCT basis of a new group-based intervention (Take Control Course; TCC) is presented, including a PCT understanding of exposure and awareness techniques. The TCC targets transdiagnostic processes and therefore contributes to emerging research regarding how a transdiagnostic theory can inform effective interventions. Initial effectiveness data for the TCC is reported in full elsewhere (Morris et al., 2015) and a RCT is currently underway. Both the initial feasibility study and the RCT recruit from primary care psychological services.

## A transdiagnostic approach

Considerable research has identified transdiagnostic maintenance processes across numerous disorders (Mansell, 2011; Nolen-Hoeksema and Watkins, 2011)^1^. Deficits in executive control, such as difficulties disengaging from a current task, are also found across a range of disorders (Schultz and Searleman, 2002; Fernández-Serrano et al., 2011). See Nolen-Hoeksema and Watkins (2011) for a more detailed summary of transdiagnostic processes.

Some interesting and promising transdiagnostic groups have already been developed; for example, Barlow and colleagues Unified Protocol has recently been delivered in a group-format and Norton's transdiagnostic anxiety groups are well-established (Norton, 2008; Wilamowska et al., 2010; Bullis et al., 2014); see Newby et al. (2015) for a meta-analysis of transdiagnostic interventions. However, these differ from the TCC in a number ways. A key difference is that none of the previous transdiagnostic interventions draw on PCT. It is advantageous to develop interventions from different theoretical perspectives so that mechanisms of change can be compared and understood (Follette and Beitz, 2003; Rosen and Davison, 2003). Other differences are described in Morris et al. (2015) in summary:

The features of TCC, that differentiate it from pre-existing interventions are: (a) basis in PCT; (b) no explicit emphasis on challenging the content of cognitions; (c) briefer group-based format; (d) explicitly flexible delivery mode; (e) broadly transdiagnostic focus that targets generic mechanisms (maladaptive/inflexible control and goal conflict). (p. 3).

One potential advantage of a transdiagnostic approach is the potential to bring us closer to understanding the mechanisms that underpin psychopathology, in light of increasing cross-disciplinary evidence for common processes (Mansell et al., 2009; Pierre, 2010; Nolen-Hoeksema and Watkins, 2011). Convergent evidence from neurobiological and cognitive-behavioral research suggests that current diagnostic categories do not precisely specify the factors that cause and maintain psychopathology (Morris and Cuthbert, 2012). For example, research has indicated that “genetic and environmental risk factors for mental illness induce susceptibility to broad domains of psychopathology, rather than discrete categorical disorders, because they disrupt core connectivity circuits in ways that necessarily produce transdiagnostic symptoms” (Buckholtz and Meyer-Lindenberg, 2012, p. 990). This has resulted in an increased drive for understandings of psychopathology based on mechanistic empirical data (e.g., Research Domain Criteria project; Cuthbert, 2015). The emphasis on understanding mechanisms that underline psychology seems a vital one in order to explain the converging empirical evidence.

There is growing empirical support for the presence of common maintenance processes, but there is much less understanding regarding which theoretical account best explains how and why these processes maintain psychological distress (Harvey et al., 2004; Mansell et al., 2009; Nolen-Hoeksema and Watkins, 2011). Traditional CBT accounts have been adapted to focus on targeting processes across disorders (Norton, 2008; Forgeard et al., 2011). However, it is argued that other accounts (such as PCT) could also offer a useful understanding of how and why numerous transdiagnostic processes maintain psychological distress across disorders. For example, PCT was initially conceptualized as transdiagnostic because it was founded as a framework for understanding psychological functioning as a whole and so the suggested mechanisms apply across all disorders (Alsawy et al., 2014). It provides a functional model of how psychopathology arises and how recovery can be facilitated (Powers, 1973). PCT is described as “functional” because PCT is a theory from which actual working systems can be constructed (see Carey, 2011, for greater theoretical discussion of this and http://www.perceptualrobots.com for some practical examples). PCT will be described in more detail in the upcoming sections.

Further, PCT is a core process account; core process accounts suggest that there are a few universal processes that underlie psychopathology (Ingram, 1990; Mansell et al., 2009). One of the challenges to transdiagnostic theories is parsimoniously explaining why a plethora of maintenance processes, such as repetitive negative thinking, perfectionism, are associated with psychopathology. Core process accounts provide a parsimonious way of integrating the relationships between these processes (see Mansell et al., 2009, for a discussion of other theoretical accounts that could integrate such findings). Empirical evidence that there are universal processes that underlie psychological dysfunction across disorders is currently small, but is increasing (Field and Cartwright-Hatton, 2008; Aldao and Nolen-Hoeksema, 2010; Bird et al., 2013a; Johnson et al., 2013). For example, a study in a mixed diagnostic group found that the variance of a range of maintenance processes was best explained by a single factor (Patel et al., 2015).

## An introduction to perceptual control theory (PCT)

The following sections introduce PCT, and summarize relevant research examining basic principles. Research supporting key aspects in clients experiencing psychological distress is also summarized. Key terms are summarized in Table 1.

**Table 1:** **Summary of key Perceptual Control Theory (PCT) terms used**.

**Term**	**Definition**	**Example**
Reference values or goal	Internal standard that is based on genetic predisposition and/or past experience.	Reference values can be considered as a set of personal “just rights”. There is a huge range of possible examples, from reference values for a good cup of coffee (e.g., milky but strong) to reference values for being a good person (e.g., kind, honest etc.).
	“*Goals” and “reference values” refer to a broad range of reference points that encompass values, beliefs, schemas etc*.	
Control	Keeping a perception as close as possible to a desired reference value.	Being a caring friend; Keeping feelings of anxiety at zero; Living a good life.
Error (or discrepancy)	The difference between the wanted and the experienced reference value.	Feeling that have let a friend down by not being caring enough; Anxiety experienced as more than can tolerate; Feeling of not living life as wanted.
Control hierarchy (system)	Internal reference values that are arranged in a hierarchical network.	Each individual will have a multitude of hierarchies. Higher-level goals (for example, the self-concept of being successful) leading to the setting of sub-goals for an individual's principles (e.g., achieving at work), which in turn regulate lower-level, shorter-term goals (e.g., working long hours).
(Goal) conflict	The state when two control systems attempt to control an experience with respect to two (or more) opposing reference values.	Carey (2008) gives this example of a client who presented with depression, indecisiveness and lack of sense of purpose. The client had a conflict between reference values of a child self that is spontaneous and reckless and a parent self that has duties. Both the “child self” and “parent self” values would often apply to the same decision or behavior.
Reorganization	When there is awareness of conflict between reference values (or error) within an individual then the reorganization process begins to make random changes. Trial-and-error changes continue until the error is reduced.	A shift in perspective, or “aha moment”, during the therapy process (or outside of therapy) could indicate that reorganization has successfully occurred (Gianakis and Carey, 2011). For example, to return to the client in the goal conflict example, once this client becomes aware of the conflict between child and parent selves they could realize one self better fits certain contexts (such as the parent self at work).

### Living is control

**Figure 1:**
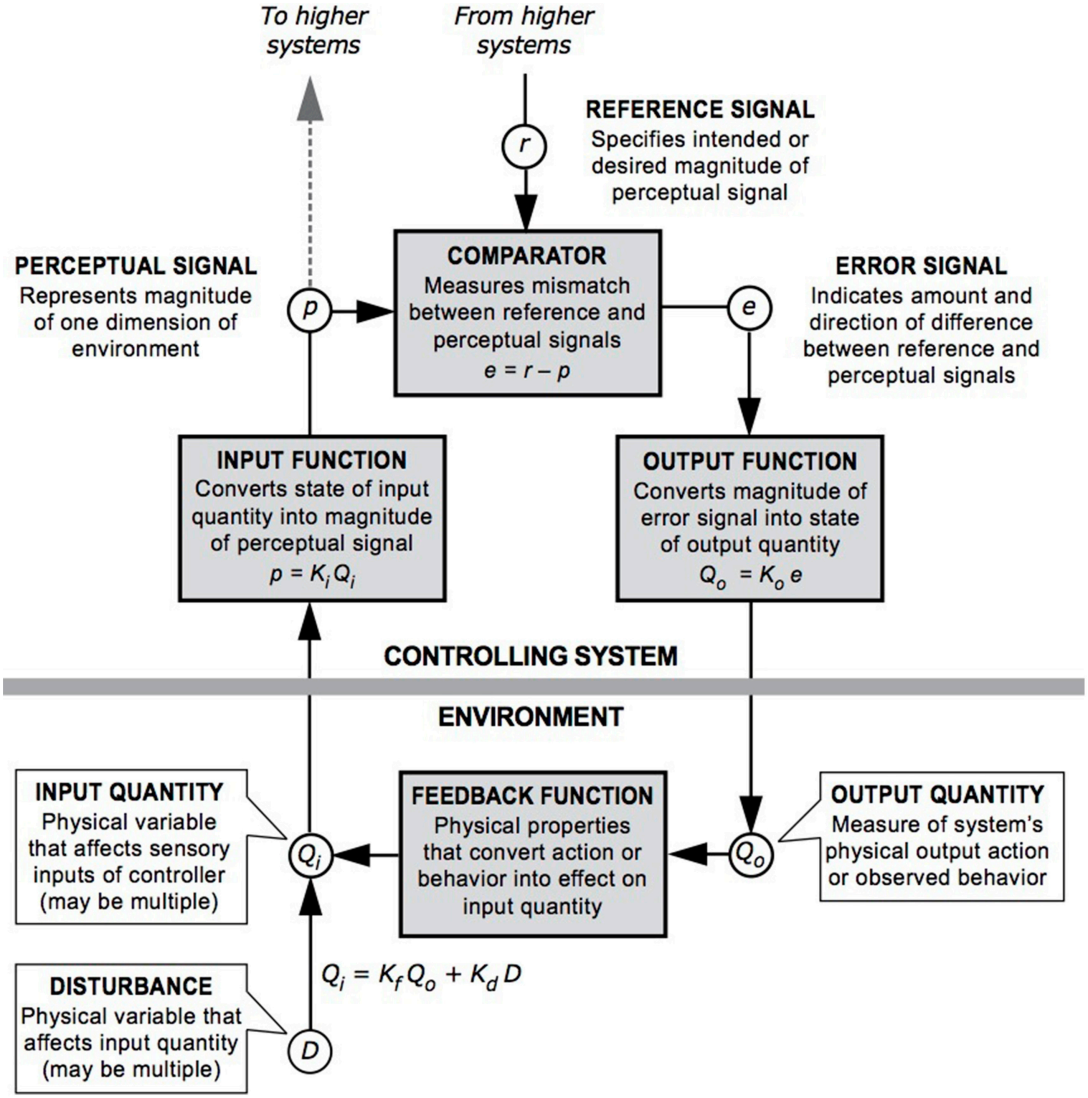
**A model of the closed negative feedback loop described in PCT; definitions of key components are included within the diagram [Redrawn by Dag Forssell from a diagram by William T. Powers]**.

The tenet that perception is controlled by behavior (rather than behavior that is controlled) is a particular emphasis of PCT (Marken and Mansell, 2013). Control is an ongoing process of comparing current perception against an internal goal or standard, and acting to minimize discrepancies. See Figure 1 for the closed “negative feedback” loop that is the basic unit of control within PCT. A full pathway round such a closed loop is necessary to implement control of perception by behavior. Even when a stimulus is subliminally presented it will seem that a stimulus triggers a response, when in fact a control system always acts against disturbances in the environment to minimize a discrepancy. For example, a socially anxious person who wishes to keep a long interpersonal distance away from other people during a conversation will be regularly moving forward or backward to maintain this distance without needing to be aware of every movement of the person they are talking to. Therefore, there is greater acknowledgment of feedback processes within PCT than in traditional stimulus response models, whereby processing a stimulus triggers an observable behavior and no feedback process to regulate goal states is explicitly implicated.

As a simplified version of the “negative feedback” loop, three essential aspects enable living beings to make events happen the way they want (control), which are *perception, comparison*, and *action* (Powers, 1973). These processes are specified precisely in terms of mathematical equations that can be used to model these relationships (Marken, 2009). *Perception* refers to perceiving the current experience or situation, *comparison* refers to assessing the current experience against an internal standard, and then the ability to *act* refers to acting on the environment to make what is being perceived to match the internal standard. Current perception is constantly compared to internal standards and actions occur to minimize any discrepancies from these standards. For example, in a noisy café a person will speak at a particular volume to make themselves heard, if a noisy group leaves they will reduce their voice so that it does not appear too loud. The reduction in volume occurs because the voice they hear *(perceive)* has become different from their standard for “acceptable volume” *(compare)* and they decrease volume to reduce the discrepancy *(act)*.

Research using computerized tracking tasks has provided evidence for the proposition that perception is controlled through action. Tasks involve participants keeping a pointer lined up with a target while the computer program randomly moves the pointer around. Such tasks require participants to keep the pointer “on target” and use their actions to dynamically alter their trajectory when the movements of the computer program take them away from their goal. A number of studies have found that what is being controlled is the perception of keeping the marker aligned rather than the behavior of moving the mouse (e.g., Marken et al., 2013; Marken, 2014). These tasks are an analog of everyday control, such as keeping a comfortable interpersonal distance or moderating the loudness of speech. Evidence supporting the tenet that perception is controlled by behavior, and other tenets of PCT, has been further reviewed elsewhere (Grawe, 2007; Pellis and Bell, 2011; Marken and Mansell, 2013).

One significant challenge to the fundamental tenet that behavior is a process of controlling perception is from studies that could indicate that behavior occurs in the absence of perception, i.e., organisms producing behavioral results without perception of these results. Such studies involve participants who do not have nerve feedback from touch and proprioception (Mechsner et al., 2007). Therefore, when visual feedback is removed, movement behaviors are conducted in the absence of the majority of the perceptual feedback normally utilized. However, it is suggested within these studies that perception of these behaviors may still be a controlled process, relying on visual imagery (internal perception) or memory of the behavior (Mechsner et al., 2007; Guillaud et al., 2011). Further research is required in order to test whether participants are producing controlled results without perception and to establish whether these studies do undermine a fundamental tenet. The interested reader is directed toward Taylor (1999) for a summary of a number of objections directed at PCT and how these can be addressed.

### Loss of control is a cause of distress

A central tenet of PCT is that well-being involves maintaining control over one's life, and, that psychological distress represents loss of this control. The associations between lack of control over external events and psychological distress have been extensively reviewed elsewhere (e.g., Chorpita and Barlow, 1998; Forgeard et al., 2011). For example, research suggests that early experience of loss of control contributes to anxiety/depression (Chorpita and Barlow, 1998; Suárez et al., 2009). Perceived loss of control of teenagers and adolescents has been shown to mediate the relationship between family environment (e.g., communication, behavioral control) and anxiety (Ballash et al., 2006; McLeod et al., 2007; Nanda et al., 2012). Research also indicates that the inability to influence internal events (e.g., thoughts, feelings, impulses) is related to psychological distress, including anxiety and depression (Dell'Osso et al., 2006; Teachman et al., 2012). Overall, substantial research supports the principle that loss of control is associated with psychological distress.

In line with this research, the “loss of control” described in PCT can be over external or internal events; this refers to a state whereby an individual's perceptions are not sufficiently close to their reference value (or desired standard). For example, they are worrying much more than they want (more than their standard for an acceptable level of worry). Whether an individual reports they have lost control, i.e., their perceived level of control, may vary. Commonly, when individuals access therapy they are aware that aspects of their experience are not as desired and this can be described in terms of “loss of control”. However, there will also be instances whereby an individual does not perceive they have lost control and they have (or vice versa); an example of the former would be someone who describes being in control of their expression of anger when they commence therapy, but later realizes that this level of anger conflicts with an important goal of maintaining a loving relationship with their partner (this understanding is further detailed in later sections on the role of conflict and awareness). The inability to influence undesired external and internal events is more likely to lead to actual (and perceived) loss of control, as individuals will be less able to bring their experience in line with their desired standard. Multiple conceptualizations of control exist within the extant literature; for example, in the mid-1990s over 100 uses of control were identified within the psychological literature (Skinner, 1996). PCT provides an operational definition of control, which is based on a functional theoretical explanation of the mechanisms of control.

One specific example of the loss of control experienced within psychopathology is loss of control of emotions, i.e., an individual perceiving that their emotional experience is not as desired. A number of accounts, including PCT, suggest that “negative” emotions arise in response to perceiving stimuli (internal or external) in order to prepare the individual for action and to meet a goal (Boudreaux and Ozer, 2013)^2^. Further, it has been suggested and evidenced elsewhere that negatively valenced emotions endure when progress toward a goal is impeded (Carver and Scheier, 1998, 2013; Powers, 2005, 2008b). In accordance with this, a PCT model specifies that these negative emotions arise when there is *error* within the control system (Carey, 2011). Error refers to the signal within the individual that something is discrepant, i.e., when a conflict between goals is experienced. Negative emotions endure when the goal is not (or cannot be) pursued and the error is not corrected (Carey et al., 2014a), i.e., at times of loss of control. Therefore, ongoing negative affect and anxiety indicate a loss of control, as they occur when error persists and goals are not obtained.

### Control is managed over many levels

A number of psychological theories have conceptualized the human mind as being organized hierarchically (e.g., Trope and Liberman, 2010). Grafton and Hamilton (2007) review varied strands of evidence, including functional imaging and computer modeling studies, to support the premise that complex motor actions are organized hierarchically with respect to distal goals. Uithol et al. (2012) review additional evidence and conclude that the explanation most consistent with the data is one that specifies dynamic interaction between the levels of the hierarchy and that “elements higher on the hierarchy are represented longer or are more stable than lower ones” (Uithol et al., 2012, p. 1083). Such a conclusion is highly compatible with a PCT account of the hierarchical structure of control.

Although this evidence for hierarchy supports a PCT account, PCT offers a distinct account of the hierarchical organization of control. For example, only PCT states that the output of each level in a hierarchy is the reference value for what the level below in the hierarchy should perceive (Powers, 1973). A higher-level goal of “being successful” could lead to the setting of sub-goals, such as “meeting work deadlines”; this in turn could lead to the setting of lower-level goals, such as “stay late to work on an important project” and to corresponding perceptual goals for motor actions. A range of simulations have used hierarchical PCT-based models, from a multi-legged robot to human arm movement, and indicate the functionality of this understanding (Kennaway, 1999; Powers, 1999, 2008a).

PCT proposes that humans have at least 11 levels of perceptual organization (Powers, 1973). The highest of these are the system concept, principle, and program perceptions. Program-level perceptions describe programs of action, such as “brushing teeth”. Principles are general rules that organize lower-level perceptions. System concepts are sets of principles that relate to more abstract values, such as self-concept and general values; for example, “being a good person” (Powers, 1973; Bird et al., 2009). System concepts may be somewhat analogous to core beliefs in CBT, and similarly principles could be related to dysfunctional attitudes (Beck, 1980; Bird et al., 2009; Dobson, 2009).

### Conflict undermines control

An important cause of loss of control within PCT is conflict (Carey et al., 2014b). Although conflicts between goals will arise frequently, circumstances in which this conflict is enduring can result in distress and psychopathology. Carey (2008) gives the example of a client with anxiety, and possible PTSD, who has a conflict between “wanting to let her daughter do normal things” and “wanting to keep her safe”. One of the ways in which wanting to keep her daughter safe manifests is a reluctance to let her go out of the house; however, it seems plausible that many parents would experience conflicts like this but would have some degree of balance and flexibility regarding meeting these goals.

Large numbers of studies support correlational and predictive relationships between elevated goal conflict and psychopathology, including relationships with depression, anxiety, and negative affect; similarly self-concordance (low goal conflict) is associated with and predictive of well-being (e.g., Brockmeyer et al., 2013; Kelly et al., 2015). Although overall the relationship between conflict and psychopathology is supported, a minority of studies has failed to replicate this, explained below.

### Conflict between higher-level goals is more detrimental than conflict between lower-level goals

A specific prediction of PCT is that chronic conflicts at higher-levels will result in greater psychopathology than conflict at lower-levels (Alsawy et al., 2014). The studies that did not replicate the findings described in the previous section (of a relationship between elevated goal conflict and psychopathology) primarily measured conflict using methods that involve participants explicitly quantifying the extent of conflict between their self-generated goals (Kelly et al., 2015). It has been suggested that the null findings are due to these measures assessing lower-level conflicts that participants have easy access to (Kelly et al., 2015). Direct evidence for the greater pathological effects of conflicts at higher-levels comes from studies that indicate that higher-level conflict is particularly problematic (e.g., Kelly et al., 2011).

It is also acknowledged that healthy individuals are able to flexibly move their awareness up and down the hierarchy to address both long-term and short-term goals, i.e., it is important to implement higher-level goals via lower-level ones (Watkins, 2011). Flexible movement of awareness entails responding to the contextual demands of different circumstances, i.e., the employment of most appropriate level within the goal hierarchy to meet the current task demands. For example, in circumstances where a relatively concrete goal is being pursued (such as, meeting a particular friend), but the goal is currently unobtainable then a higher-level focus (e.g., having social contact) will be more adaptive than a lower-level focus because this enables greater flexibility. Watkins (2011) provides a useful account of circumstances in which different levels of goal identification are predicted to be adaptive/maladaptive. However, he focuses on the dimension of abstraction, whereas PCT would suggest that different levels of goals can be functionally different on other dimensions. A greater level of abstraction often characterizes higher-levels, but there are other differences, such as the highest levels taking longer to process than the lowest levels (Marken et al., 2013).

### Control without awareness can exacerbate conflict

As mentioned above, measures of implicit goal conflict are more consistently related to psychopathology than measures of explicit goal conflict (Kelly et al., 2015). A PCT account proposes that this is because without awareness of conflicted goals, i.e., when goals are implicit and less consciously accessible, conflicts will not be resolved. When a reference value (or goal) is controlled for without taking into account another reference value that is also controlling this experience, this is described as *arbitrary control* (Powers, 1973). For example, after a traumatic accident an individual might experience sleep problems and flashbacks and want to discuss this with others. However, they might struggle to talk about these experiences due to a reference, which they are not aware of, “other people will think I'm crazy”. Without awareness of this reference their experience is likely to be of loss of control and by not discussing their problems an important goal is blocked.

Due to the importance of awareness in resolving goal conflict, and meeting important goals, processes that limit such awareness will maintain psychopathology. Therefore, repeated and prolonged employment of processes (e.g., worry and rumination), which limit attention and disrupt awareness of goal conflict, promote on-going difficulties (Watkins, 2008). It should be noted that it is the extent to which these processes prevent the achievement of other goals that determines whether these are maladaptive. There is some evidence that processes/strategies that maintain psychopathology (such as, rumination or suppressing emotions) are applied more rigidly than “adaptive” strategies (Aldao and Nolen-Hoeksema, 2012). Further, to focus on the example of rumination, research has found that a form of “rumination”^3^ can have adaptive consequences (Watkins, 2008; Ottaviani et al., 2013). Evidence suggests that it is when rumination involves abstract evaluative thoughts regarding the self and emotions, particularly when it is negatively valanced, that it is maladaptive (Nolen-Hoeksema et al., 2008; Watkins, 2008). Arguably it is this type of “rumination,” rather than a non-evaluative conceptual focus on present experience, that is more likely to limit awareness and constitute arbitrary control. This can be compounded when an individual feels unable to control their thinking (Morris and Mansell, in preparation; Teachman et al., 2012; Ottaviani et al., 2013). This understanding corresponds with the wider literature on control and self-regulatory theories (e.g., Pyszczynski et al., 1987; Carver and Scheier, 1998), although a PCT account differs in some important respects (the emphasis on the role of conflict, specification of a reorganization mechanism). Cognitive and behavioral inflexibility has been associated with psychopathology in a number of studies across disorders (Kashdan and Rottenberg, 2010).

### Change is spontaneous but its focus is guided by awareness

Change is often conceptualized as a linear process that occurs over a certain number of therapy sessions; for example, clients are often offered a set contract (e.g., 6 or 12 sessions) and are assessed for symptom reductions at the end of these. However, there is accumulating evidence that change does not always occur in such a predictable way with evidence that clients can experience sudden gains (sometimes described as insights, eureka moments etc.), early in therapy, that can account for a significant amount of their improvement (Aderka et al., 2012).

Therefore, a change mechanism is required that would enable diverse experiences of change. The proposed change mechanism is known as *reorganization*. When there is a loss of control (or error) within the control system then the reorganization process begins to make random changes, until the error is reduced (Powers, 1973). The reorganization system responds to error, it does not require volitional control. It functions in the same way as other homeostatic processes within the human body; for example, an optimal internal temperature is maintained within our bodies without us having to think about it. These changes occur at the point that awareness is directed within the control system. The clinical implications of this include explaining the findings that sudden gains can result in lasting symptom change because these would be examples of successful reorganization. The functional effects of this model, i.e., whether such a process leads to effective change, have been tested and confirmed using computer simulations (Marken and Mansell, 2013). Support for the proposal that reorganization is activated by error in the system is provided by research showing that unconsciously activated goals are more likely to reach awareness when goal progress is obstructed (i.e., when conflict) occurs (Dijksterhuis and Aarts, 2010).

### Shifting awareness to the systems driving enduring higher-level conflicts is the key to recovery

Within PCT, it is proposed that it is vital for long-term recovery that reorganization occurs at the source of the goal conflict(s) (Alsawy et al., 2014). Reorganization occurs at the point that awareness is directed and, therefore, bringing awareness to enduring conflicts is vital to recovery (Marken and Carey, 2014).

Initial support for the proposal that reorganization promotes psychological change, and “follows” awareness, comes from research demonstrating that greater awareness of conflict and higher-level processes during a psychological intervention predicted greater distress reduction and problem resolution (Gaffney et al., 2013). In a clinical sample accessing PCT-based individual therapy, research has found that participants' scores on a self-report measure of reorganization processes was negatively associated with depressive symptoms and increased following therapy, effect size *d* = 1.11 (Bird, 2013).

According to PCT, the primary reason for using awareness/mindfulness techniques, i.e., techniques that develop sustained non-reactive attention, is to develop a “window” of awareness that can be brought to bear on conflicted goals. Effective therapies use various methods to shift and sustain awareness on sources of goal conflict (Carey, 2011; Higginson et al., 2011). Given that this is a shared aspect of psychological therapies, targeting this aspect will resemble facets of other therapies; for example, motivational interviewing promotes awareness of competing goals (Miller and Rose, 2009). But the explicit emphasis on processes, such as control, hierarchy, and reorganization, in the TCC result in differences (see “Components of the TCC”). There is convergent evidence that techniques that involve shifting and sustaining awareness lead to significant psychological change (Carey, 2011; Fjorback et al., 2011). It is beyond the scope of this paper to give a comprehensive account of theories of exposure and mindfulness.

An individual CBT therapy has been developed based on PCT; called Method of Levels (MOL) (Carey, 2006). This aims to help clients to become aware of higher-level goals and facilitate the process of reorganization, in order to resolve goal conflicts (Carey et al., 2009). There have been a number of evaluations of MOL in routine clinical practice trials. These have shown significant post-treatment reductions in symptoms on standardized measures of psychological distress, with moderate to large effect sizes, for clients experiencing a range of problems including depression, anxiety disorders and anger issues (Carey and Mullan, 2007, 2008; Carey et al., 2009, 2013). A pilot RCT was conducted in primary care (Bird et al., 2013b) where MOL was compared to control (waiting list or regular CBT treatment). Significant improvements were demonstrated on ratings of anxiety and depression in both conditions at follow-up, with larger improvements in the MOL condition for ratings of anxiety. Although a promising individual treatment, a MOL approach cannot be used with large groups. TCC is informed by MOL and includes some similar treatment components, such as a curious questioning style and an explicitly flexible delivery style.

### Interim summary

In summary, PCT gives a functional explanation of how psychopathology develops across disorders and also indicates how this can be resolved. As would follow from this, causal accounts of psychopathology based on conflict between higher-level goals are found across a variety of presentations, such as obsessive-compulsive disorder (Pitman, 1987), PTSD (Carey et al., 2014b), depression (Hyland, 1987), bipolar disorder (Mansell, 2010), addiction (Webb et al., 2010), and dissociative disorders (Johnson, 2009; Mansell and Carey, 2012). In the next section, the potential service delivery advantages of a transdiagnostic approach are explored, and the ways in which PCT informs the TCC are described.

## Development of the take control course (TCC)

Studies have indicated that as much as half of those seeking treatment for mental health problems access primary care services alone (Kessler et al., 2005), highlighting the importance of cost-effective and accessible interventions in these contexts (Alexander et al., 2010). Although a substantial proportion of clients accessing primary care have problems with depression and/or anxiety, clients with a range of presenting problems are treated (Glover et al., 2010; Barkham et al., 2012) and PCT offers an understanding of psychopathology relevant to this range. TCC was designed to be offered within primary care contexts. Given that PCT is transdiagnostic theory it is hypothesized that TCC could be adapted for other client groups. For example, if offering the TCC to clients with addictive disorders a number of changes could be made. One change could be to the focus of the session on “what blocks our control” (Session 2) and this could focus on control of urges to partake in substances, e.g., by prioritizing other goals and increased awareness, rather than on repetitive thinking (Webb et al., 2010; Fujita, 2011; Thomsen, 2015). Although PCT is a transdiagnostic theory, this does not mean that there cannot be differences in the likely content of goal conflicts within different presenting problems. However, because such presenting problems would still be underpinned by self-regulation difficulties resulting from goal conflict, much of the intervention content used to address these conflicts would be similar.

Transdiagnostic groups are reviewed elsewhere (Morris et al., 2015). Although initial efficacy and effectiveness data for transdiagnostic groups is promising (e.g., Norton and Philipp, 2008), the groups available involve a significant time commitment (e.g., 10 weekly 2-h sessions; Maia et al., 2013; Newby et al., 2015). However, there is considerable evidence that briefer interventions are effective for clients with mild to moderate anxiety or depressive disorders (e.g., Newman et al., 2010; Richards and Borglin, 2011). Therefore, the TCC offers a briefer format (6 weekly sessions lasting an average of 1 h) and has the potential to be offered to large groups. Evidence that psychological change can be achieved in primary care through large group courses (White and Keenan, 1990; White, 1998; Kitchiner et al., 2009) suggests that such groups should not rely on participant disclosure and discussion. The TCC format relies on experiential exercises, worksheets, videos, and facilitator presentations. However, using the current TCC delivery model (where groups range from 5 to 15) some discussion is possible if participants wish. Distinctive features were described previously, and Table 2 illustrates how the PCT basis results in differences in the ways that principles are conceptualized and employed therapeutically.

**Table 2:** **Key principles of PCT and examples of similarities/differences to familiar psychotherapies**.

**Principle/process**	**Therapy**			
	**Take Control Course (based on PCT)**	**“Second wave” CBT^a^**	**Acceptance and Commitment Therapy (based on RFT)**	**Mindfulness-based Cognitive Therapy (using ICS theoretical perspective)^b^**
Control	Control as fundamental to life; functional process to be restored and refined [see *Table 1* for precise definition]	Not emphasized, except in some specific models [e.g., Hofmann's (2007) social anxiety model; Fairburn et al.'s (1999) Anorexia model]	Control as problematic (especially regarding internal processes); to be reduced in therapy	Attentional control seen as functional and to be increased
Goal conflict	Higher-level goal conflict key source of distress and psychopathology [see *Table 1* for definition]	Not explicitly emphasized, although approach-avoidance conflicts are identified in some models (e.g., Kimbrel, 2008)	Not explicitly targeted, but potentially addressed through acceptance focus	Not emphasized
Hierarchy	Goals are organized in a complex and many layered hierarchy; 11 layers have been specified [see *Table 1* for definition]	Core beliefs, dysfunctional attitudes, strategies are organized hierarchically. These represent distorted and dysfunctional cognitions rather than goals	Values and specific goals are specified, but hierarchical organization not specified	Nine interacting cognitive subsystems are proposed, some of which are hierarchically arranged; these are specialized for handling a particular type of information
“High-level goals” or “values” identified	Pertinent higher-level goals identified through sustained awareness on problems [see *Table 1* for definition]	Not explicitly emphasized. Concrete goals for therapy are seen as important, but often not specified in the models used	Values identified through value clarification exercises	Not explicitly emphasized, but can be discussed in regards to relapse prevention
Reorganization (and observable indicators of this process)	Provides a mechanistic account of how change happens, indicated by shifts in perspective/ “insight” moments [see *Table 1* for definition]	Cognitive reappraisal and schematic change could be observable indicators (but not mechanistically specified)	Not specified	Modification of affect-related schema, e.g., reducing the likelihood that mild depression will regenerate depressogenic schematic models, could be observable indicator
Awareness	An index of the current focus; reorganization occurs at the focus of current awareness	Not emphasized	Targets cognitive defusion and acceptance	Targets attention regulation, acceptance, decentering/reperceiving (similar to defusion) etc.

*PCT, Perceptual Control Theory; RFT, Relational Frame Theory; ICS, Interacting Cognitive Subsystems*.

*The purpose of this table is not to provide a comprehensive comparison of therapies. It is focused on PCT-relevant processes; for example, cognitive biases are not included because, while these are fundamental to CBT, they are only significant in a PCT conceptualization to the extent they cause chronic higher-level goal conflict*.

a *Second wave CBT refers to the models that emerged during and after the fusion of cognitive and behavioral therapies in the 1970s (Rachman, 1997)*.

b *There is not a universally agreed theoretical basis for Mindfulness-based interventions. Interacting Cognitive Subsystems (ICS) has been proposed as a theoretical account of Mindfulness-based Cognitive Therapy (Teasdale et al., 1995) and is included as it provides a detailed explanation of the ways in which interactions between cognitive and affective processes contribute to psychological distress*.

### Protocol development

The main development team for the TCC consisted of a Clinical Psychologist (and academic), Mental Health Nurse (and academic), Trainee Clinical Psychologist, and three Gateway Workers (experienced mental health professionals). In addition, frequent development meetings were held with a range of other clinicians who worked, or had worked in primary care settings; with further suggestions from a specialist in graphic media and a number of academics working on PCT related research. After the initial consultation and development phase, a pilot with eight clients attending the service was run of the first TCC session in order to obtain detailed section-by-section feedback. The first session was chosen for the pilot because it introduces a number of recurring themes and techniques. Some sections were revised, or replaced, based on participant feedback. Formative feedback was collected during the delivery of all subsequent sessions.

### Components of the TCC (including the rationale based on PCT)

In accordance with the evidence that change happens at a different pace for different people there is a strong emphasis on client self-direction and flexible control within the TCC (see sections on “Conflict between higher-level goals is more detrimental than conflict between lower-level goals” and “Change is spontaneous but its focus is guided by awareness” for background, or Morris et al., 2015 for a more detailed summary). Furthermore, increasing evidence indicates that offering clients flexibility in attending psychological treatments increases effectiveness for services and is valued by clients (Carey and Spratt, 2009; Carey et al., 2013). It has been suggested, that in clinical practice clients regulate their ongoing treatment attendance and continue treatment until they have achieved a “good enough” level of change (Barkham et al., 2006; Carey et al., 2013). Providing flexibility of attendance and engagement could promote attendance by promoting control and mitigating the impact of internal conflicts that would block attendance (e.g., fears about emotional exposure; Schauman and Mansell, 2012; Murphy et al., 2014).

Flexibility is offered in other ways. Specific homework tasks are not given, but at the end of each session participants reflect on what they are taking from the session and can set themselves a task. Sessional evaluation forms are filled in and session content can be adapted in response to feedback. For example, although the core content of each session is prescribed, certain techniques re-occur and so later implementation of techniques can be adapted in accordance with feedback. Furthermore, the emphasis on experiential rather than didactic learning is employed to promote individual clients to consider the specific ways they can apply techniques to themselves.

Each session has a theme and includes videos of clients who have accessed either previous interventions within the service or earlier TCC. These provide opportunities for the group to hear accounts of other people's experiences of distress and recovery, as relevant to the session theme. Some opportunities are provided for participants to share their experience, but it is strongly emphasized that this is not a requirement.

In accordance with the PCT theoretical account, targeted throughout the TCC are: (1) understanding the process of control, including the degree to which one can, and desires, control over various aspects of one's life; (2) awareness of valued higher-level goals (and reasons for change); (3) awareness of higher-level goal conflict; (4) encouragement of flexible ways to control and reduction of processes (including rules, habits, routines, and mental processes), that block valued goals. Clients are expected to gain a greater understanding of the aspects of their life that they can control, and ways of increasing control in these areas. Further, the TCC enables clients to consider the goals that are most important to them and provides ways of supporting them to achieve these in a variety of contexts. The following is a description of key components of the six sessions.

#### Session one: thinking about control

Control is the basis of this course. The overall theme is to encourage participants to clarify what responses get in the way of them achieving the things that are important to them and to consider the things that they can, and want to, have more control over. For example, one exercise uses a “continuum of control” to facilitate participants to identify how much control they have over different aspects of their life. This group exercise often promotes discussion regarding the difference between how much control people think they have and how much control they actually have. Other session components begin to offer techniques that promote sustained attention on problematic experiences that are indicative of goal conflict. These include an awareness exercise, which encourages clients to stay with an experience and notice how this is different from judging (or thinking about) that experience; and a goal-focused exposure exercise that involves imaginal exposure to an uncomfortable experience (e.g., social situation, a recurrent worry, feeling of failure), but not one that will be extremely uncomfortable (Carryer and Greenberg, 2010). Different forms of both goal-focused exposure and awareness techniques can be offered in nearly all of the sessions. Awareness techniques precede exposure components to facilitate enhanced attention prior to exposure.

#### Session two: what blocks our control? addressing negative thinking: self-critical voice, worry and dwelling on things

A key message is that individual thoughts, even negative ones, are not a problem. Even worry, rumination and self-criticism are processes that many people engage in without any adverse effects (Watkins, 2008). They are a problem only to the extent that they get in the way of important life goals, or they limit a person's awareness of important goals and goal conflicts. Therefore, more repetitious and prolonged negative thinking can be problematic. Videos and metaphors are used to delineate potentially more problematic thinking processes, e.g., recurrent negative worry and rumination. Strategies are taught to help address thinking processes that block control. For example, a form of problem-solving that emphasizes potential goal conflict is offered as an alternative to thinking over the same thing repeatedly.

#### Session three: feeling in control short-term vs. getting control of your life

The principle that goals are organized hierarchically provides a key basis of this session. A fundamental message of the session is that sometimes (not always) our attempts to control our emotions, avoid distress or keep safe can actually get in the way of us doing things that are really important to us. This encourages clients to focus on their longer-term (higher-level) goals and to consider prioritizing these over short-term goals that are getting in the way. For example, a common short-term goal that can prevent achievement of long-term goals is “I must avoid all distress/anxiety”. One of the early components is a group exercise that emphasizes the normality and functionality of emotions. Later in the session, participants complete a worksheet to weigh up whether examples of things that they do have short-term (e.g., make them feel better) or long-term benefits (e.g., move them toward their goals); then consider if there is anything they would like to change.

#### Session four: taking control of the things around you

A unique focus of PCT is that we are designed to control our environment for our own purposes. There are many things in our environment that we can change in order to make our world more like we want it to be. However, there are also some things that, objectively, we will have limited control over, such as other people. The control continuum is re-employed to consider how much control participants have over aspects of their environment that are causing them problems. An important focus of the session is consideration of control in interpersonal scenarios, e.g., a discussion or argument. The importance of clarification of *higher-level* goals during interpersonal scenarios, especially goals that might be shared, is explored using examples; it is suggested that these processes can help navigate arguments and discussions etc. For example, if someone had a higher-level goal of “having peace of mind”, as well as a lower-level goal of “getting my partner to clean up more” awareness of the higher-level goal could allow greater flexibility in how the lower-level goal can be achieved and could reduce the tendency to get caught in the detail of an argument. Expressing a potentially shared higher-level goal, e.g., valuing the relationship, could further reduce the escalation of conflict by increasing shared ground. There is evidence from sociological work that interpersonal conflict escalates quicker than it is resolved (McClelland, 2004), hence the importance of a flexible perspective to reduce initial disagreement. Furthermore, studies have found that shared reference values are important to reduce interpersonal conflict (McClelland, 2004).

#### Session five: building on strengths, qualities, and resources

The focus of the session is to encourage participants to recall the strengths, qualities and resources they have, especially at times when things are difficult for them. The rationale behind this session is that self-concept goals are higher-level ones (Mansell and Carey, 2009). Psychopathology can arise when another reference value remains in conflict with a self-concept reference value; for example, “I must be strong” vs. “I am weak and inadequate” (Tai, 2009). An example of a metaphor, used in this session, is that of a tree, which needs water and sunlight and similarly people need resources to grow. Another imagery-based technique used in this session is participants creating an image that represents qualities that they value and believe will support them.

#### Session six: moving forward: what gets me stuck? what helps?

A general overview of TCC is provided. Clients are encouraged to choose elements that they want to revisit. They complete a worksheet regarding the things that are helping them feel in control and signs that they are struggling. This worksheet has similarities to a “relapse prevention” session in a traditional CBT approach. Allowing choice in the material covered, and encouraging clients to reflect on what works for them is intended to encourage flexible control. In this context, flexible control refers to the ability to move awareness up and down the control system in order to meet valued goals.

### Recurrent intervention components

As aforementioned, certain components are repeated within the TCC. These components include exposure, awareness/mindfulness, and metaphor/imagery. The PCT basis of exposure, awareness/mindfulness is detailed below. Although these components are found within other CBT approaches, the PCT basis results in a specific way of utilizing and framing these that is distinct from other approaches. However, work is ongoing to examine the mediating effects of mechanisms according to PCT.

#### Imaginal goal-focused exposure

According to PCT, exposure is perceptual and not behavioral. This means that people attempt to keep the properties of their experience (e.g., closeness, intensity) within a desired range using dynamic changes in behavior (e.g., movement, using substances; Brady and Raines, 2009; Carey, 2011). The proposed problem is that people have conflicting perceptual goals; for example, the conflict between avoiding a past memory of a distressing assault, with the need to experience this memory to manage similar situations in the future. This leads to loss of control as the person oscillates from one goal to the other. According to PCT, exposure is designed to help people hold this perceptual conflict in mind and establish a new organization of goals (reorganization) that allows them to regain overall control. The conflict between personally relevant goals will be most emphasized if clients are able to direct the exposure process (Brady and Raines, 2009), with some guidance. Guided self-direction best promotes change, because if pressure from the therapist is too great the client's awareness will be less directed to their underlying conflicted goals (and more to the conflict created by the therapist's pressure).

Therefore, in the TCC exposure is framed in such a way to ensure it is self-directed, guided by client's life goals and promotes sustained attention on conflicts (Carey, 2011). Consequently, it is described as “imaginal goal-focused exposure.” The way in which the exercise is framed is always in the context of important higher-level goals in order to promote awareness of goal-conflict, instead of promoting habituation to anxiety, emotional-processing, or reappraisal of a situation (e.g., Foa et al., 2006). These outcomes may arise, but they are not seen as the reason for exposure.

The majority of empirical studies have found that greater perceived control during exposure reduces anxiety and distress levels; however, findings are not totally consistent (Mcglynn et al., 1994; Rose et al., 1995). In one of the studies in which greater perceived control did not reduce anxiety the average exposure durations chosen by participants were very brief, which supports the notion that some therapist direction is useful (Craske et al., 1991). A number of theorists, such as Gray and McNaughton, have highlighted the key role of goal conflict in activating an anxiety response (e.g., Hirsh et al., 2012). A recent study, which compared the performance of students with high and low levels of spider phobia, found that high spider phobic individuals responded in a way suggestive of goal conflict (Oliver and Mansell, 2013).

#### Awareness/mindfulness

From a PCT perspective, awareness is important in a number of ways. It is important, that awareness of higher-level conflicted goals that are causing psychopathology is encouraged, in order to resolve these conflicts (Carey, 2011). According to PCT, the primary reason for using awareness techniques is to develop present moment awareness that can be brought to bear on conflicted goals. Therefore awareness techniques used in the TCC are shorter than those delivered in other approaches; for example, MBCT (Fjorback et al., 2011). Use of awareness techniques support clients to sustain attention on problematic experiences indicative of goal conflict (e.g., during the imaginal goal-focused exposure). It has been suggested that a range of effective therapies enable clients to sustain attention on problem-relevant experiences for longer than would otherwise occur (Carey, 2011). Through this sustained attention on such experiences the individual's perception is likely to become more elaborate, and inherent conflicts more likely to be resolved.

It is also important that individuals are able to flexibly move awareness through the control hierarchy in order to reduce the likelihood of psychopathology and achieve long-term goals (Watkins, 2011). Individual differences in the general level of goal focus maintain symptoms of a number of psychopathologies; for example, a general focus on lower-level goals can lead to greater impulsivity (Fujita and Carnevale, 2012). Emerging research indicates that greater mindfulness is associated with increased clarity regarding important goals and greater flexibility of goal pursuit (Crane et al., 2010, 2012). Enhanced selective and executive attention can be developed through short-term meditation practice and may increase flexibility of awareness; however, it is not clear whether the amount of practice during the TCC would be sufficient (Chiesa et al., 2011). However, TCC participants are provided with support to practice awareness techniques at home if they choose to. Further, even very brief mindfulness conditions have been shown to reduce reactivity to repetitive thoughts (Levin et al., 2012).

## Summary and conclusions

Research at psychological process and neurobiological levels indicates the promise of transdiagnostic accounts in understanding and treating psychopathology (Nolen-Hoeksema and Watkins, 2011; Morris and Cuthbert, 2012). PCT provides a functional transdiagnostic model of how psychopathology arises and how recovery can be facilitated.

There are other transdiagnostic models available. These include Relational Frame Theory (RFT; Hayes et al., 2006), the Self-Regulatory Executive Dysfunction (S-REF) model (Wells and Matthews, 1996; Wells, 2007), and Interacting Cognitive Subsystems (ICS; Barnard and Teasdale, 1991). It is beyond the scope of this review to provide a detailed comparison of these models (see Mansell et al., 2009, for a more detailed account of this area). Establishing which model is the most accurate will require ongoing empirical testing. Specifically it would be useful to directly compare the purported mechanisms of different models in the same study. Further, there may be differences in terminology within different theories that obscure conceptual similarities. For example, it could that the “process” described as awareness, mindfulness and detached mindfulness in different theories refers to the same underlying process, and that its benefits arise from similar mechanisms. Although it is important to be precise and accurate in terminology, this should not come at the cost of integrating potentially very similar constructs.

Further research is required to directly test the relative contribution of some of the PCT mechanisms predicted to underlie psychopathology; for example, additional research is required into whether higher-level conflict is a more significant contributor to psychopathology than lower-level conflict. Another important area will be examining the extent to which the PCT basis of TCC facilitates its effectiveness; for example, the extent that the resolution of higher-level goal conflict predicts successful exposure. This would facilitate detailed comparison with other explanatory theories and interventions.

In summary, this article has presented the theoretical basis and design of a transdiagnostic intervention based on PCT. While some components of this intervention could be familiar, it is focused around the concept of control and based on the premise that control is exercised at various levels for a human being to continue living. Psychological distress is regarded as the manifestation of loss of control, typically caused by prolonged conflict between high-level goals. Therefore, change is promoted by shifting awareness to this conflict and sufficiently sustaining awareness in order to allow spontaneous changes that resolve the goal conflict and restore control. The TCC provides the tools for individuals to apply these principles in an accessible, succinct manner.

## Author contributions

LM, WM, and PM all substantially participated in the development of the intervention described with this manuscript. LM and WM primarily wrote the review manuscript, with PM also contributing to the conceptualization and content. All authors were involved in revision of the manuscript for important intellectual content.

## Funding

This manuscript was primarily written whilst the first author was in receipt a North West Doctoral Training College, Economic Social Research Council, CASE Studentship Award.

### Conflict of interest statement

The authors declare that the research was conducted in the absence of any commercial or financial relationships that could be construed as a potential conflict of interest.

## Footnotes

^1^Transdiagnostic maintenance processes are cognitive and behavioral processes that contribute to the maintenance of symptoms of psychological distress across a wide range of psychological disorders (Harvey et al., 2004).

^2^The term “negative” is used here to distinguish emotions (e.g., anger, fear, and depression) from emotions commonly described as pleasant and that do not promote the same tendency toward action (e.g., contentment, equanimity) (Powers, 2008b).

^3^Sometimes rumination is defined in terms of repetitive negative, abstract, self-focused thinking and this construct is associated with depression and negative affect Nolen-Hoeksema et al., 2008. However, it is noted that in clinical settings clients do not always describe this experience with such specificity. It is more common to talk about experiences, such as “dwelling on things,” which can refer to a broader processes of self-reflection that may have adaptive as well as malaptive functions.
